# Histones and heart failure in diabetes

**DOI:** 10.1007/s00018-018-2857-1

**Published:** 2018-06-22

**Authors:** Veera Ganesh Yerra, Andrew Advani

**Affiliations:** Keenan Research Centre for Biomedical Science and Li Ka Shing Knowledge Institute of St. Michael’s Hospital, 6-151, 61 Queen Street East, Toronto, ON M5C 2T2 Canada

**Keywords:** Epigenetics, Complications, Acetylation, Methylation, Cardiovascular disease, Post-translational modifications

## Abstract

Although heart failure is now accepted as being a major long-term complication of diabetes, many of the recent advances in our understanding of the pathobiology of diabetes complications have come about through the study of more traditional microvascular or macrovascular diseases. This has been the case, for example, in the evolving field of the epigenetics of diabetes complications and, in particular, the post-translational modification of histone proteins. However, histone modifications also occur in human heart failure and their perturbation also occurs in diabetic hearts. Here, we review the principal histone modifications and their enzymatic writers and erasers that have been studied to date; we discuss what is currently known about their roles in heart failure and in the diabetic heart; we draw on lessons learned from the studies of microvascular and macrovascular complications; and we speculate that therapeutically manipulating histone modifications may alter the natural history of heart failure in diabetes.

## Introduction

Over the past decade, there has been a growing appreciation that the old dogma of diabetes complications being either microvascular or macrovascular, although convenient, was, in fact, wrong. Aside from the classical complications of nephropathy, retinopathy, and neuropathy, and of coronary artery disease, cerebrovascular disease, and peripheral vascular disease, people with diabetes are also at increased risk of certain cancers [1], cognitive impairment [2], bone fractures [3], and heart failure [4]. The recognition of these previously overlooked complications coupled with a maturing understanding of the pathogenesis of classical complications has led to a realization that the end-organ effects of diabetes often represent a continuum rather than discrete pathological entities. The increased prevalence of heart failure in diabetes exemplifies this continuum [4].

Paralleling our evolving understanding of the continuum of end-organ injury in diabetes, has been an evolving understanding of the pervasive roles that epigenetic processes may play in the development of diabetes complications [5, 6]. The relationship between epigenetic processes and diabetes complications has historically been investigated in the setting of microvascular (reviewed in Refs. [7, 8]) or macrovascular (reviewed in Ref. [9]) diseases, reflecting the historical dichotomous classification. In contrast, the contribution of epigenetic dysregulation to heart failure in diabetes has been comparatively overlooked. Epigenetic processes play pivotal roles in cardiac development and in the development of heart failure in other settings [10], and it seems likely, therefore, that they play similarly pivotal roles in the development of heart failure in diabetes. The two best characterized epigenetic processes are DNA methylation and the post-translational modification of histone proteins and the epigenetic processes that, to date, have revealed themselves to be most amenable to therapeutic intervention are histone protein modifications. Here, with the goal of facilitating research in this generally understudied area, we summarize the best characterized histone modifications and their enzymatic writers and erasers; we review the evidence that histone protein modifications contribute to the development of heart failure; and we draw on lessons learned from the studies of microvascular and macrovascular diseases to consider how changes in histone proteins may affect the development of heart failure in diabetes.

## Heart failure in diabetes: scope of the problem

Diabetes mellitus currently affects over 451 million people across the globe [11] and a wealth of epidemiological evidence indicates that these people are at an increased risk of developing heart failure. In the Framingham cohort, for example, the incidence of congestive heart failure was increased approximately 2.4-fold in men with diabetes and 5.3-fold in women with diabetes [12]. Likewise, in a retrospective cohort study of over 17,000 individuals with Type 2 diabetes or individuals without diabetes of similar age and sex, the incidence of congestive heart failure was more than doubled at 30.9 cases per 1000 person-years (compared to 12.4 cases per 1000 person-years for people without diabetes) [13]. Even though the incidence of heart failure increases with age, the excess risk conferred by comorbid diabetes is much higher in people of younger age, with an increased relative risk of 11-fold in individuals under the age of 45 years in comparison to 1.8-fold in individuals aged 75–84 years [13]. However, heart failure risk is not limited to individuals with Type 2 diabetes. People with Type 1 diabetes are also at increased risk. For instance, in a recent prospective case–control study of 33,402 individuals with Type 1 diabetes and 166,228 matched controls, the hazard ratio (HR) for hospitalization for heart failure was 4.69 (95% confidence interval [CI] 3.64–6.04) [14]. For people with heart failure, the presence of comorbid diabetes increases all-cause mortality (HR 1.28 [95% CI 1.21, 1.35]) and risk of hospitalization (HR 1.35 [1.20, 1.50]) [15]. Moreover, not only does the presence of heart failure in diabetes portend a particularly poor prognosis, but it is also vastly expensive. In one model, congestive heart failure was the most expensive incident cost in a population of 10,000 adults with diabetes, estimated at an annual expected cost of $7,320,287USD [16]. In short, for people with diabetes, heart failure is prevalent, it is expensive, and it carries an, especially, poor prognosis.

People with diabetes are at increased risk of both heart failure with reduced ejection fraction (HFrEF; ejection fraction (EF) < 50%) and heart failure with preserved ejection fraction (HFpEF; EF ≥ 50%). In 2012, in USA, approximately 5.8 million individuals (2.4% of the population) had heart failure and, in the community, approximately 50% of heart failure cases are caused by HFpEF [17]. Approximately 40% of people with HFrEF and 45% of people with HFpEF have diabetes [18], but the outlook for people with diabetes and heart failure is poor regardless of the EF [19]. For instance, in the Candesartan in Heart failure: Assessment of Reduction in Mortality and Morbidity (CHARM) programme, both all-cause death and hospitalization were increased in people with diabetes in comparison to people without diabetes (all-cause death per 1000 patient years, diabetes 116.3, no diabetes 72.9, *p* < 0.001; first hospital admissions per 1000 patient years, diabetes 473.4, and no diabetes 327.2, *p* < 0.001) [20]. In that study, rates of cardiovascular death were 119.1 per 1000 patient years for individuals with diabetes and HFrEF and 58.6 per 1000 patient years for individuals with diabetes and HFpEF [20]. However, diabetes was associated with a greater risk of cardiovascular death or hospitalization for heart failure in people with HFpEF than it was in people with a reduced EF (HFpEF and diabetes, HR 2.0 [1.70–2.36]; HFrEF and diabetes, HR 1.60 [1.44–1.77]; interaction test, *p* = 0.0009) [20].

## Causes of heart failure in diabetes

**Fig. 1:**
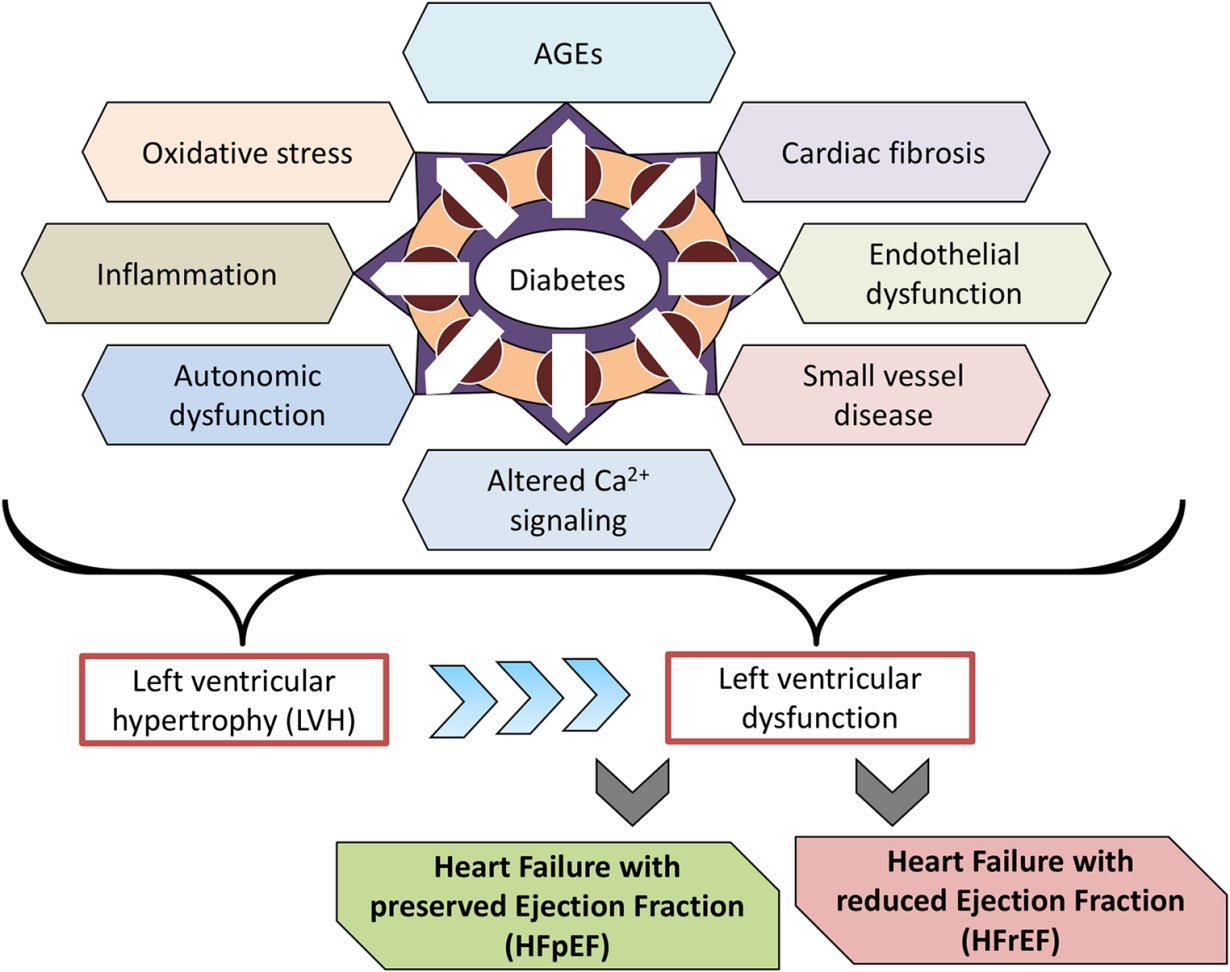
Cellular processes affected by diabetes and its comorbidities that affect cardiac cell function and that can predispose to heart failure development

In 1972, investigators reported post-mortem findings of heart failure in four individuals with diabetes but without coronary artery disease [21]. This led to the coining of the term “diabetic cardiomyopathy” used to describe myocardial dysfunction in a person with diabetes, but in the absence of hypertension or coronary artery disease [22]. However, the term itself remains imperfectly defined and the extent to which elevated blood glucose levels alone can cause myocardial dysfunction remains controversial. For example, one study that combined functional, biochemical, and morphological techniques failed to identify evidence of myocardial dysfunction in 185 persons with Type 1 diabetes, with a mean duration of diabetes of over 20 years but without coronary artery disease or hypertension [23]. Nonetheless, the increased prevalence of heart failure in diabetes is undisputed and just as the term “diabetic kidney disease” is now favored over “diabetic nephropathy”, so “heart failure in diabetes” may be preferred over “diabetic cardiomyopathy”, attesting to the multifactorial nature of the condition and the common coexistence of predisposing comorbidities. With respect to these predisposing comorbidities, the prevalences of both coronary artery disease [24] and hypertension [25] are increased in people with diabetes and diabetes exacerbates all forms of cardiovascular disease [26]. At a molecular level, hyperglycemia and accompanying formation of advanced glycation end products (AGEs), endothelial dysfunction, impaired calcium homeostasis, oxidative stress, inflammation, abnormalities in glucose and fatty acid utilization, autonomic dysfunction, myocardial fibrosis, and small vessel disease act in concert to impair myocardial function in diabetes (reviewed in Refs. [27–29]). This manifests as left ventricular hypertrophy (LVH) and adverse remodeling, ultimately impairing systolic and/or diastolic function [26] (Fig. 1). Indeed, electrocardiogram (ECG) or echocardiogram evidence of LVH signifies a high risk for the development of heart failure [30] and in the Look AHEAD trial approximately 5% of overweight or obese individuals with Type 2 diabetes had ECG evidence of LVH [31].

## Heart failure in diabetes and its relationship to current treatments

### Heart failure treatments

Conventionally, the treatment of heart failure in people with diabetes has been the same as for people without diabetes. Therapy for heart failure includes the use of renin–angiotensin system blockers such as angiotensin converting enzyme (ACE) inhibitors, angiotensin receptor blockers (ARBs), or an angiotensin receptor–neprilysin inhibitor (ARNI), aldosterone receptor antagonists, β-blockers, diuretics, combination of hydralazine and isosorbide dinitrate, ivabradine, and digoxin if indicated and device therapy with an implantable cardioverter defibrillator or cardiac resynchronization therapy where appropriate [32, 33]. The evidence supporting the use of these agents derives from the studies of individuals with HFrEF, and unfortunately, similar evidence of their benefit in individuals with HFpEF is lacking [32, 34].

### Anti-hyperglycemic agents and their relationship to heart failure in diabetes

The relationship between heart failure risk and agents used to lower blood glucose levels in people with Type 2 diabetes has recently been reviewed [26]. Briefly, blood glucose-lowering therapies may worsen heart failure, have neutral effects, or, in some cases, actually improve outcomes in patients. For instance, the thiazolidinediones, rosiglitazone, and pioglitazone have both been associated with an increased risk of admission to hospital with heart failure [35, 36]. With respect to heart failure outcomes, incretin-based therapies [i.e., glucagon-like peptide-1 (GLP-1) receptor agonists or dipeptidyl peptidase-4 (DPP-4) inhibitors] may be largely neutral [37–41], although the DPP-4 inhibitor, saxagliptin, was associated with an unexpected increase in the risk of hospitalization for heart failure in the Saxagliptin Assessment of Vascular Outcomes Recorded in Patients with Diabetes Mellitus (SAVOR)—Thrombolysis in Myocardial Infarction (TIMI) 53 trial (HR 1.27, [95% CI 1.07–1.51]) [42]. Similarly, in the Examination of Cardiovascular Outcomes with Alogliptin versus Standard of Care (EXAMINE) trial, the number of participants hospitalized for heart failure was numerically greater, albeit non-significantly, with the DPP-4 inhibitor alogliptin than with placebo (3.1 vs. 2.9%; HR 1.0 [95% CI 0.79–1.46]) [43]. Unfortunately, for older blood glucose-lowering agents (i.e., metformin, sulphonylureas, and insulin), there are insufficient data to draw robust conclusions as to safety, benefit, or harm. This is because these agents were in clinical use for many years prior to the 2008 U.S. Food and Drug Administration Guidance for Industry that mandated the demonstration of the absence of cardiovascular harm for anti-hyperglycemic agents used in the treatment of Type 2 diabetes [44].

In 2015, the results of the EMPA-REG OUTCOME trial surprised many in the diabetes clinical care and research communities by demonstrating a significant reduction in cardiovascular events in people with Type 2 diabetes at high cardiovascular risk treated with the sodium–glucose cotransporter 2 (SGLT2) inhibitor, empagliflozin [45]. In that study, treatment with empagliflozin was associated with a significant reduction in the primary outcome of major adverse cardiovascular events (HR 0.86 [95.02% CI 0.74–0.99], *p* = 0.04 for superiority), a 38% relative risk reduction for death from cardiovascular causes, a 32% relative risk reduction for death from any cause, and a 35% risk reduction for hospitalization for heart failure (2.7 vs. 4.1%) [45]. In the Canagliflozin Cardiovascular Assessment Study (CANVAS) Program, hospitalization for heart failure was similarly reduced with the SGLT2 inhibitor canagliflozin (HR 0.67 [95% CI 0.52–0.87]), although there was an increased risk of amputation (HR 1.97 [95% CI 1.41–2.75]) [46]. In both EMPA-REG OUTCOME [47] and CANVAS [46], over 85% of participants had no prior history of heart failure, and thus, the beneficial effects of SGLT2 inhibition may be viewed as being those of heart failure *prevention* rather than necessarily heart failure *treatment*. Indeed, there is a relative paucity of data on the efficacy of SGLT2 inhibitors in patients with established heart failure, with or without diabetes, and there is a similar paucity of data as to whether SGLT2 inhibitors are equally effective in patients with HFpEF and HFrEF [26]. Furthermore, even in the impressive EMPA-REG OUTCOME study, treatment with empagliflozin, on top of standard-of-care, and thus arguably representing current best practice, did not negate heart failure, only reducing it, hospitalization for heart failure still occurring at a rate of 9.4/1000 patient years [45]. As the global prevalence of diabetes increases over the coming years (expected to exceed 693 million people by 2045 [11]), there is thus a pressing need to explore innovative new treatment opportunities. One such opportunity may be to exploit the post-translational modification of histone proteins and the effects that these changes can have on gene transcription and cellular (dys)function.

## Histone protein post-translational modifications and the enzymes that control them

**Fig. 2:**
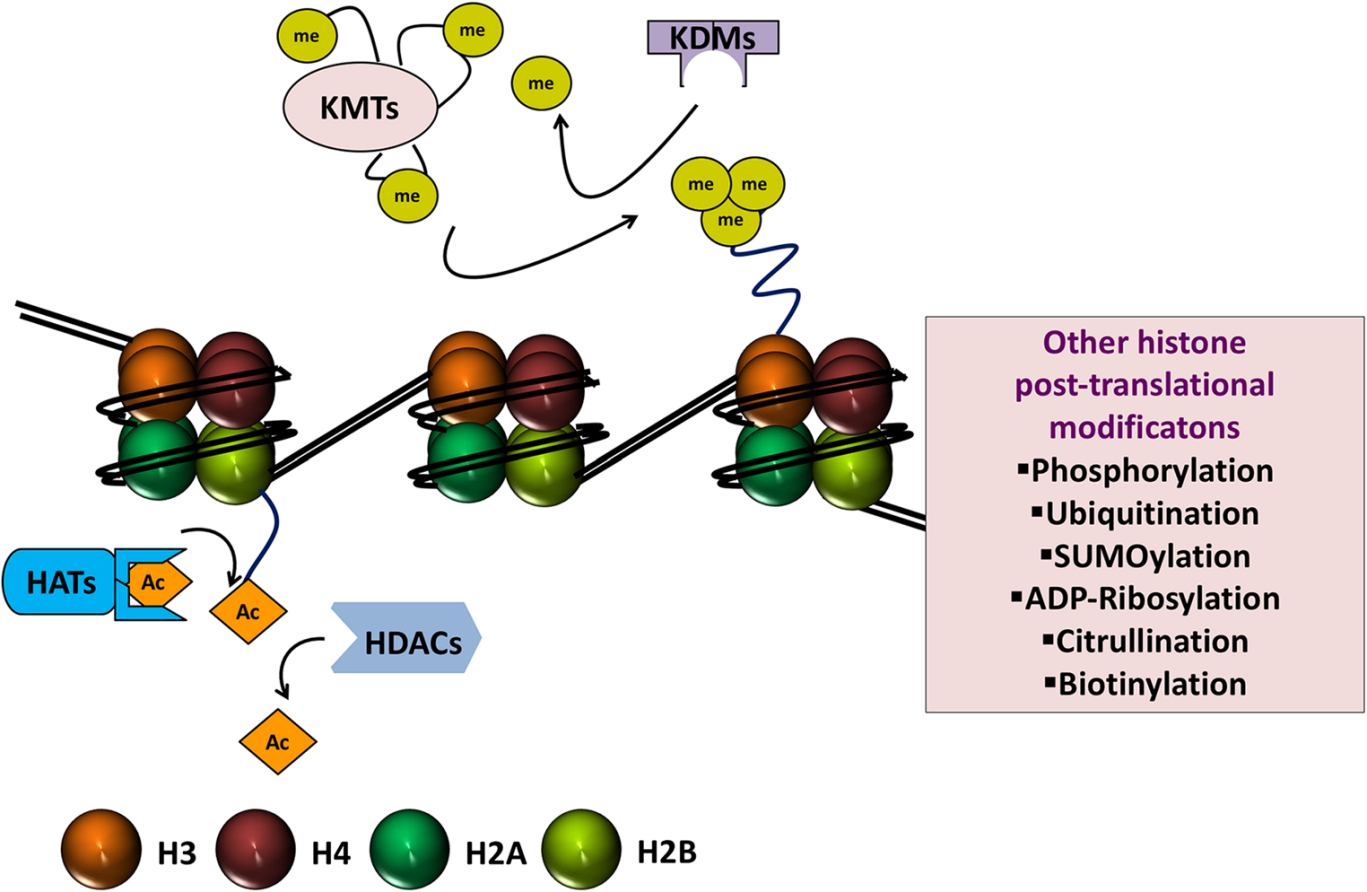
Nucleosome core particle, histone proteins, and histone writers and erasers. The nucleosome is made up of approximately 146 bp of DNA coiled nearly twice around an octamer of histone proteins, made up of two copies each of histone H2A, histone 2B, histone H3, and histone H4. The protruding amino tails of histone proteins can undergo post-translational modifications that affect the expression of genes in close proximity. Illustrated are the major histone post-translational modifications that have been studied in the context of heart failure and diabetes (i.e., acetylation and deacetylation and methylation and demethylation), along with their enzymatic writers and erasers, histone acetyltransferases (HATs or KATs, where K = lysine), histone deacetylases (HDACs), histone methyltransferases (HMTs or KMTs), and lysine demethylases (KDMs). Other post-translational modifications that can affect histones and consequently gene expression but that have been comparatively less well studied in heart failure and diabetes are shown in the boxed area

Histones are the basic protein building blocks of chromatin and, together with DNA, they make up the fundamental unit of chromatin, the nucleosome core particle. A nucleosome core particle consists of approximately 146 bp of DNA wrapped nearly twice around a histone octamer which itself is made up of two copies each of the four core histone proteins H2A, H2B, H3, and H4 (Fig. 2) [48]. Nucleosomes are joined together by stretches of linker DNA. H1 linker histones, whilst not being part of the nucleosome core particle, bind linker DNA at the entry and exit sites of the nucleosome and help to stabilize the whole complex [49]. In comparison to the core histone proteins, the function of linker histones has been relatively less well characterized [50]. The packaging of DNA into nucleosomes and hence chromatin enables it to exist in a very condensed fashion, essentially providing a means whereby the approximate 2 m length of a DNA strand can fit inside a eukaryotic nucleus that is typically less than 10 µm in diameter. This packaging is important for how changes in histone proteins can influence the expression of nearby genes. Histone proteins have long, protruding amino tails that are susceptible to a range of post-translational modifications and these modifications can facilitate gene activation or repression. Histone protein post-translational modifications include acetylation, methylation, phosphorylation, ubiquitination, SUMOylation, ADP ribosylation, citrullination, and biotinylation [51]. By far and away, the best characterized of these modifications are histone-lysine acetylation and deacetylation and histone-lysine methylation and demethylation, and thus, these modifications are the focus of the current treatise.

### The nomenclature of histone writers and erasers

The acetylation or deacetylation of lysine residues on histone proteins is regulated by two opposing groups of enzymes: histone acetyltransferases (HATs) and histone deacetylases (HDACs), and the methylation or demethylation of lysine residues on histone proteins is regulated by histone methyltransferases and lysine demethylases. The discovery of these enzymes resulted in nomenclature systems that are somewhat non-coherent and also inconsistent between species, and in an effort to rectify this situation, in 2007, scientists proposed a new nomenclature [52]. Thus, HATs are also referred to as KATs, lysine methyltransferases as KMTs, and lysine demethylases as KDMs, where K = lysine [52]. At the time of the new nomenclature, the authors did not propose renaming the HDAC family as they felt that this classification was coherent enough [52]. However, authors will occasionally refer to HDACs as KDACs (e.g., [53]), recognizing that histones are not the only substrates of many lysine residue-modifying enzymes.

### Mechanisms through which histone modifiers and histone modifications facilitate gene activation or repression

Histone modifications can influence gene transcription by several mechanisms that can be broadly classified into two categories: (1) by altering chromatin compaction (a direct effect); and (2) by influencing the recruitment of effector complexes (indirect effects) [51, 54, 55]. Histone modifications can affect chromatin compaction by altering the electrostatic charge relationship between histone proteins and DNA. This can be the case for histone acetylation or phosphorylation. For instance, by neutralizing the charge on lysine residues, acetylation can weaken the association between positively charged histone proteins and negatively charged DNA, thus improving the accessibility of protein machinery to target sites of DNA. The indirect effects of histone protein modifications on effector recruitment and retention are, however, more pervasive in terms of their influence on gene transcription. These indirect effects influence the actions of chromatin-modifying enzymes, transcription factors, and other histone protein modifiers and modifications. For example, histone modifications can facilitate the recruitment or regulate the efficiency of ATP-dependent chromatin remodeling enzymes (e.g., SWitch/Sucrose Non-Fermentable (SWI/SNF) [56]) that enhance the accessibility of nucleosomal DNA, playing an important role in the regulation of transcription by RNA polymerase II [57]. The enzymes responsible for histone protein post-translational modification are often co-recruited to their target sites in protein complexes together with transcription factors [58], and histone modifications can influence the recruitment of transcription factors at epigenetically distinct transcriptional start sites, proximal promoter regions, and distal enhancers [59–61]. Alternatively, by blocking the access of remodeling complexes to chromatin, certain histone protein modifications limit gene transcription [54]. They can also influence the recruitment of other histone modifiers, and consequently, the formation of other histone modifications [62]. For example, the HAT Gcn5 can acetylate lysine residue 14 on histone H3 (H3K14) more effectively when serine residue 10 on histone protein H3 (H3Ser10) is phosphorylated [63]. Finally, certain histone modifications are mutually exclusive. For example, H3K4 methylation generally marks actively transcribed genes and is associated with H3K9 hypomethylation. This arrangement may be mediated by the physical and functional association of the H3K4 methyltransferase mixed lineage leukemia or myeloid/lymphoid leukemia 2 (MLL2) and the H3K9 demethylase Jumonji domain-containing 2B (JMJD2B) [64].

Histone protein modifications also function in tandem with the other major category of epigenetic processes, DNA methylation. Classically, methylation of DNA has been studied in the context of its occurrence at the 5-position of cytosine residues (referred to as 5-methylcytosine, or 5mC) most commonly located within regions of DNA known as CpG islands [65, 66]. These are regions of DNA arbitrarily greater than 200 bp in length that contain phosphodiester-linked cytosine and guanine residues occurring at a frequency of more than 50%. Methylation of CpG islands at transcriptional start sites serves as a recognition point for reader proteins, such as methyl CpG binding protein 2 (MECP2), which block gene transcription [67]. Accordingly, in this context, DNA methylation is generally considered to facilitate transcriptional repression. However, DNA methylation can also occur away from transcriptional start sites, including in gene bodies, and at these sites, DNA methylation may not be associated with gene repression but may alternatively be involved in gene activation (by the preclusion of repressor binding sites) or in alternative splicing [68]. More recently, it has been recognized that gene transcription may also be regulated by the oxidative formation of 5-hydroxymethylcytosine (5hmC, DNA hydroxymethylation), a process that is regulated by the ten–eleven translocation (TET) family of 5mC dioxygenases, which is commonly (although not exclusively) associated with transcriptional activation [69–73]. Histone protein modifications and DNA methylation are commonly interrelated, one epigenetic mark influencing the generation of the other and vice versa [74]. This bidirectional relationship can be mediated by direct interactions between histone modifications and DNA methylating enzymes or between sites of DNA methylation and histone-modifying enzymes. For example, the DNA methyltransferase Dnmt3a possesses an interacting domain that links DNA methylation to unmethylated H3K4, whereas the H3K4 methyltransferase MLL1 contains a CpG-interacting domain that could link H3K4 methylation to unmethylated DNA [75].

Aside from their effects on histone proteins, many ostensibly histone-modifying enzymes can also modify non-histone proteins, especially transcription factors. These modifications can influence gene expression by affecting transcription factor stability and function. This is the case for both lysine (de)acetylating and lysine (de)methylating enzymes. For example, the enzyme SET7/9 methylates histone proteins, transcription factors, and other epigenetic regulators. In its role as a histone methyltransferase, SET7/9 monomethylates H3K4 [76]. However, several transcription factors have also been found to be substrates for SET7/9 (e.g., p53, E2F1, interferon regulatory factor 1 (IRF1), forkhead box O3 (FOXO3), p65, retinoblastoma protein (pRb), and signal transducer and activator of transcription 3 (STAT3), amongst others) [77, 78]. Furthermore, SET7/9 also methylates other enzymes that can exert their own epigenetic effects, including Dnmt1 [79] and the HAT, p300/CBP-associated factor (PCAF) [80], affecting their enzymatic activities. Thus, when interpreting the effects of histone-modifying enzymes, one should remain cognizant that these effects may be both chromatin-dependent and chromatin-independent.

In summary, histone-modifying enzymes or histone modifications rarely, if ever, function in isolation to regulate gene transcription. Rather, a conceptual framework is beginning to emerge whereby functional genomic regions are tagged by specific histone marks that support the transcriptional response to stimuli, histone modifications functioning synergistically with each other, with other epigenetic regulators and with canonical transcription factors.

### Histone acetylation

Utilizing acetyl CoA as a cofactor, HATs catalyze the transfer of acetyl groups to the ε-amino group of the lysine side chain of histone proteins, which neutralizes the histone protein’s positive charge and weakens the interaction between histones and DNA. This causes chromatin to adopt a more open conformation that favors transcriptional activation. By removing acetyl groups from lysine residues, HDACs restore the positive charge of histone tails leading to chromatin condensation which should favor transcriptional repression [81]. In reality, however, the situation is more complex than this. For instance, histone acetylation may also serve as a recognition point for bromodomains [55, 82], which are modules of approximately 110 amino acids in length commonly present in many proteins that associate with chromatin [82].

HATs can be grouped into five subfamilies: the Gcn5-related *N*-acetyltransferase (GNAT) superfamily, MYST [named after its first four members: MOZ, Ybf2 (Sas3), Sas2, and Tip60], p300/CBP (CREB-binding protein), transcription factors, and nuclear receptor cofactors (Table 1). There are at least 18 different HDAC enzymes, which are subdivided phylogenetically into four classes. Classes I, II, and IV are zinc-dependent histone deacetylases and comprise: Class I (HDACs 1, 2, 3 and 8), Class IIa (HDACs 4, 5, 7 and 9), Class IIb (HDACs 6 and 10), and Class IV (HDAC11). Class III HDACs, the sirtuins, are NAD^+^-dependent and, in mammals, comprise at least seven members (SIRTs 1–7) (Table 2). Both HATs and HDACs are recruited to target promoter regions as parts of large protein complexes and both classes of enzymes exhibit poor specificity for the lysine residues that they acetylate or deacetylate [83].

**Table 1:** Histone acetyltransferase (HAT) subfamilies and enzymes in humans

Subfamily	Enzyme examples	Alternative nomenclature	Histone residues acetylated
GNAT superfamily	GCN5	KAT2	H3 (H4, H2B)
	PCAF	KAT2B	H3, H4
	HAT1	KAT1	H2AK5, H3, H4
	ELP3	KAT9	H3
MYST	Tip60	KAT5	H2A, H3, H4
	MOZ	KAT6A	H3, H4
	MORF	KAT6B	H2A, H3, H4
	HBO1	KAT7	H3, H4
	MYST1	KAT8	H4
p300/CBP	p300	KAT3B	H2A, H2B, H3, H4
	CBP	KAT3A	H2A, H2B, H3, H4
Transcription factors	TAF_II_250		H3, H4
	TFIIIC90	KAT12	H2A, H3, H4
	CLOCK	KAT13D	H3, H4
Nuclear receptor cofactors	SRC1	KAT13A	H3, H4
	ACTR	KAT13B	H3, H4
	TIF2	KAT13C	H3, H4

Adapted from Refs. [52, 166, 167]

*GNAT* Gcn5-related *N*-acetyltransferases, *KAT* K-acetyltransferase, *PCAF* p300/CBP-associated factor, *HAT1* histone acetyltransferase 1, *ELP3* elongator protein complex 3, *MOZ* monocytic leukemia zinc finger protein, *MORF* monocyte leukemia zinc finger protein-related factor, *HBO1* histone acetyltransferase binding to ORC-1, *CBP* CREB-binding protein, *CLOCK* clock circadian regulator, *SRC1* steroid receptor coactivator-1, *TIF2* transcriptional mediators/intermediary factor 2

**Table 2:** Classes of histone deacetylase enzymes in humans

Class	Enzymes
Class I	HDAC1, HDAC2, HDAC3, HDAC8
Class IIa	HDAC4, HDAC5, HDAC7, HDAC9
Class IIb	HDAC6, HDAC10
Class III	SIRT1, SIRT2, SIRT3, SIRT4, SIRT5, SIRT6, SIRT7
Class IV	HDAC11

Adapted from Ref. [168]

*HDAC* histone deacetylase, *SIRT* silent mating-type information regulation 2 homolog

Numerous proteins, other than histones, can be lysine acetylated or deacetylated by HATs and HDACs and, as already discussed, the terms HATs and HDACs are somewhat of misnomers. For instance, one study seeking to gauge the extent of the lysine acetylome identified 1750 acetylated proteins in MV4-11 cells [84], whereas the HDAC, HDAC6 are localized almost exclusively in the cytosol [85]. It is, perhaps, unsurprising then that many of the studies linking lysine acetylation to either cardiac development or the (patho)physiological response to cardiac stress have demonstrated that the pivotal actions of HATs or HDACs are mediated by their acetylation or deacetylation of non-histone proteins. For instance, mutation of the Class II HDACs, HDAC5, and HDAC9 results in embryonic or perinatal lethality with variable penetrance accompanied by ventricular septal defects and a thin-walled myocardium, possibly due to superactivation of the transcription factor, myocyte enhancer factor 2 (MEF2), which is known to interact with Class II HDACs and control cardiomyocyte differentiation [86, 87]. However, the specific acetylation of histone proteins is also important to cardiac development and function. For example, the master cardiac transcription factor, GATA4, which is required for both cardiac development [88, 89] and in the adult heart [90], drives gene expression by stimulating histone H3 lysine 27 acetylation (H3K27ac) [91].

### Histone methylation

Lysine residues can be acetylated or methylated, but they cannot be both. In contrast to histone acetylation, histone methylating (and demethylating) enzymes can be remarkably specific. Histone protein methylation is catalyzed by histone methyltransferase enzymes and can occur on either lysine or arginine residues. Over 30 human proteins have histone methyltransferase activity. Amongst the histone methyltransferase family, the PRMT enzymes catalyze the methylation of arginine residues, whereas the SET family of proteins catalyze the methylation of lysine residues [92]. Lysine residues may be mono- (me1), di- (me2), or tri- (me3) methylated, whereas arginine residues may be either mono- or di- methylated [93]. Histone-lysine methyltransferases (KMTs) catalyze the transfer of a methyl group from *s*-adenosylmethionine (SAM) to ε-amino groups on lysine residues of histone tails [94], and RMTs catalyze the transfer of methyl groups from SAM to ω-guanidino nitrogen atoms on arginine residues in eukaryotes [94]. Table 3 summarizes common histone-lysine methylation marks, their effects on gene transcription, and the methyltransferases responsible for their generation.

**Table 3:** Histone-lysine methylation marks, their enzymatic writers, and their effects on gene transcription

Histone-lysine methylation mark	Function	Enzymatic writers (histone methyltransferases)
H3K4	Transcriptional activation	MLL1/KMT2A, MLL2/KMT2B, MLL3/KMT2C, MLL4/KMT2D, MLL5/KMT2E, SET1A/KMT2F SET1B/KMT2G, ASH1/KMT2H, SET7/9/KMT7 SMYD1/KMT3D, SMYD2/KMT3C, SMYD3, SETMAR, PRDM9/KMT8B
H3K9	Heterochromatin formation/transcriptional repression	SUV39H1/KMT1A, SUV39H2/KMT1B, EHMT2/G9a/KMT1C, EHMT1/GLP/KMT1D, ESET/SETDB1/KMT1E,
H3K27	Transcriptional repression	EZH1/KMT6B, EZH2/KMT6A
H3K36	Transcriptional activation	SETD2/KMT3A, NSD1/KMT3B, SYMD2/KMT3C, SETMAR, NSD2/KMT3G, NSD3/KMT3F, SETD3
H3K79	Transcriptional activation	DOT1L/KMT4
H4K20	Transcriptional repression/DNA damage response	SET8/KMT5A, SUV4-20H1/KMT5B, SUV4-20H2/KMC5C

Adapted from Refs. [6, 52, 169]

*MLL* mixed lineage leukemia or myeloid/lymphoid leukemia, *KMT* K-methyltransferase, *SMYD* SET and MYND domain-containing, *SETMAR* SET domain and mariner transposase fusion protein, *PRDM* PR domain, *SUV39H* suppressor of variegation 3–9 homolog, *EHMT* euchromatic histone-lysine *N*-methyltransferase, *GLP* G9a-like protein, *ESET* ERG-associated protein with SET domain, *SETDB* SET domain bifurcated, *EZH* enhancer of zeste homolog, *SETD* SET domain-containing, *NSD* nuclear receptor-binding SET domain protein, *DOT1L* DOT1-like histone-lysine methyltransferase

Until the relatively recent discovery of specific lysine demethylating enzymes, it was considered that histone methylation was stable and irreversible. However, it is now known that this is not the case. There are two main classes of histone-lysine demethylating enzymes (Table 4). Lysine-specific demethylases (LSDs) utilize flavin adenosine dinucleotide (FAD) as a cofactor. The best studied LSD, LSD1, functions with protein complexes such as the CoREST (restin corepressor) complex to reverse H3K4 and H3K9 methylation [95–97]. The JmjC domain-containing proteins are the most recently discovered and the largest class of histone demethylases. They catalyze histone-lysine demethylation by utilizing Fe(II) and α-ketoglutarate as cofactors [98]. Arginine demethylating enzymes have also been described [99], although, in comparison, the effects on gene transcription are more prevalent for histone-lysine demethylation.

**Table 4:** Histone-lysine demethylases and substrates

Enzyme	Alternative name	Substrate demethylated
LSD1	KDM1A	H3K4me1/2, H3K9me1/2
LSD2	KDM1B	H3K4me1/2
NO66		H3K4me1/2/3, H3K36me2/3
JHDM1B/FBXL10	KDM2B	H3K4me3, H3K36me1/2
JARID1A/RBP2	KDM5A	H3K4me2/3
JARID1B/PLU-1	KDM5B	H3K4me1/2/3
JARID1C/SMCX	KDM5C	H3K4me2/3
JARID1D/SMCY	KDM5D	H3K4me2/3
JHDM2A	KDM3A	H3K9me1/2
JHDM2B	KDM3B	H3K9me1/2/3
JHDM2C	KDM3C	H3K9me1/2
JMJD2A/JMJD3A	KDM4A	H3K9me3/2, H3K36me2/3
JMJD2B	KDM4B	H3K9me2/3, H3K36me2/3
JMJD2C/GASC1	KDM4C	H3K9me2/3, H3K36me2/3
JMJD2D	KDM4D	H3K9me2/3
PHF2	KDM7C	H3K9me1/2, H3K27me1/2
PHF8	KDM7B	H3K9me1/2, H4K20me1
JHDM1D	KDM7A	H3K9me1/2, H3K27me1/2
UTX	KDM6A	H3K27me2/3
JMJD3	KDM6B	H3K27me2/3
JHDM1A/FBXL11	KDM2A	H3K36me1/2

Adapted from Refs. [52, 169–171]

*LSD* lysine-specific histone demethylase, *KDM* K-demethylase, *JHDM* jumonji C domain-containing histone demethylase, *FBXL* F-box and leucine-rich repeat protein, *JARID* jumonji/ARID domain-containing protein, *RBP2* retinoblastoma-binding protein 2, *SMCX* Smcx homolog, X chromosome, *SMCY* SMC homolog, Y chromosome, *JMJD* jumonji domain-containing, *GASC1* gene amplified in squamous cell carcinoma 1, *PHF* PHD finger protein, *UTX* ubiquitously transcribed X chromosome tetratricopeptide repeat protein

As an illustration of the specificity of histone-lysine methylation, the H3K4 and H3K36 marks are associated with transcriptional activation, whereas the H3K9 and H3K27 marks are associated with transcriptional repression [100]. H3K4 monomethylation (H3K4me1) is commonly found at enhancer elements, whereas H3K4 trimethylation (H3K4me3) is enriched at active promoters [101]. H3K4me3 sites differ markedly between failing hearts and normal hearts both in Dahl salt-sensitive rats and in humans with congestive heart failure [102]. The histone methyltransferase protein complex responsible for H3K4 trimethylation associates with a ubiquitously expressed nuclear factor, termed pax transcription activation domain-interacting protein (PTIP) [103]. The murine model of hypertrophic heart failure induced by transverse aortic constriction (TAC) surgery is commonly employed to examine the effects of epigenetic changes on cardiac dysfunction, and mice lacking PTIP have diminished global H3K4me3 levels and exhibit a maladaptive response to TAC [104]. This maladaptive response is characterized by cardiac dilatation, decreased left ventricular function, cardiac fibrosis, and increased cell death [104]. The JmjC domain-containing histone demethylase JMJD2A catalyzes the demethylation of di- and tri- methylated H3K9 (H3K9me2/3) and di- and tri- methylated H3K36 (H3K36me2/3). Mice with cardiac-specific inactivation of JMJD2A have been reported to have an attenuated hypertrophic response to pressure overload induced by TAC, whereas JMJD2A overexpressing mice have an exaggerated response [105].

### Histone phosphorylation, ubiquitination, SUMOylation, ADP ribosylation, and glycosylation

Histone proteins may be phosphorylated on hydroxyl groups of serine, threonine, and tyrosine residues. Kinases catalyze the addition of phosphate groups from ATP and phosphatases catalyze their removal. Because phosphate groups confer a negative charge, phosphorylation, in general, is associated with open chromatin and thus facilitates gene transcription [106]. The kinase, calcium/calmodulin-dependent protein kinase II δ (CaMKIIδ) plays a key role in pathological cardiac hypertrophy [107]. It has been suggested that nuclear CaMKIIδ facilitates chromatin remodeling by phosphorylating H3Ser10, promoting the transcription of genes responsible for the hypertrophic response [108, 109].

Histone modifications can also involve the addition or removal of large bulky groups, specifically ubiquitination and SUMOylation. Ubiquitination entails the addition of the large polypeptide, ubiquitin by the sequential action of E1-activating, E2-conjugating, and E3-ligating enzymes, and the sites and degree of ubiquitination depend upon the enzyme complexes involved [110]. The addition of small ubiquitin-l modifier (SUMO) proteins also involves E1, E2, and E3 enzymes, and functions to prevent acetylation or ubiquitination at the same sites [111]. ADP ribosylation and glycosylation may also occur on amino acid residues on histone tails, although the extent to which these modifications may affect gene expression is relatively unexplored [112, 113]. There is also a comparative paucity of information pertaining to these other post-translational histone modifications and their relationship to heart failure.

## Epigenomic organization and the generation of developmental stage- and disease-specific epigenetic signatures

Recently, efforts have been made to develop comprehensive epigenomic roadmaps in experimental and human heart disease. These approaches have combined chromatin immunoprecipitation (ChIP)-sequencing for multiple different histone marks together with transcriptional analysis determined after RNA-sequencing with or without ascertainment of DNA methylation and hydroxymethylation patterns by whole-genome bisulfite sequencing and 5hmC-sequencing [114, 115]. In 2013, Papait and co-workers studied cardiomyocytes isolated from mice subjected to TAC, combining data from ChIP-sequencing for seven different histone marks (H3K9ac, H3K27ac, H3K4me3, H3K79me2, H3K9me2, H3K9me3, and H3K27me3) and RNA-sequencing data. Taking this approach, they identified a particular epigenetic signature that regulated the promoter activity of 325 of 1109 genes in experimental hypertrophic heart failure that was characterized by the mutual exclusion of histone modifications that mark areas of transcriptional activation (H3K9ac, H3K27ac, H3K4me3, and H3K79me2) and repression (H3K9me2, H3K9me3, and H3K27me3) [114]. They also identified more than 9000 possible active enhancers associated with experimental cardiac hypertrophy [114]. More recently, Gilsbach and colleagues set out to examine the epigenomic signature of human cardiomyocytes during development, postnatal maturation, and in chronic heart failure. The investigators purified cardiomyocyte nuclei from human cardiomyocytes and subjected the samples to whole-genome bisulfite sequencing, 5hmC-sequencing, ChIP-sequencing for seven histone marks (H3K27ac, H3K9ac, H3K36me3, H3K4me1, H3K4me3, H3K9me3, and H3K27me3), and RNA-sequencing for nuclear gene expression. They reported that prenatal development and postnatal maturation are characterized by active CpG methylation and histone marks at *cis*-regulatory and genic regions, but that, in heart failure, there are changes in active histone marks without major changes in CpG methylation. These active histone marks were H3K27ac, H3K4me3, H3K4me1, H3K9ac, and H3K36me3 [115]. The findings highlight the close interrelationship between DNA methylation patterns and histone modifications, and underscore the paradigm that, once they are established, DNA methylation patterns are highly stable, whereas histone modifications are more labile [74]. The derivation of epigenomic blueprints such as these may aid the development of future treatments, for instance, by facilitating the generation of cardiomyocytes from embryonic stem cells or induced pluripotent stem cells or by facilitating the epigenomic reprogramming of cells into cardiomyocytes in vivo [115].

## Histone protein modifications and heart failure in diabetes

In Table 5, we have summarized some of the most salient studies of the roles of histone proteins in heart failure. Of these 22 studies, 6 have focused on the roles of histone protein post-translational modifications in diabetes and they are elaborated upon here. One of the first reports describing alterations in histone protein modifications in the diabetic heart examined global histone changes in the hearts of uninephrectomized Type 2 diabetic db/db mice and reported an overall change in the cardiac histone signature in diabetes (i.e. increased levels of H3K23ac, H3K9ac, H3Ser10 phosphorylation, and H3K4me2 and reduced levels of H3K9me2) [116]. In the context of our current understanding of the complexity of epigenomic organization, the significance of overall global changes in these histone marks is uncertain. Concurrently, experiments in the H9c2 rat cardiomyocyte cell line have suggested that high glucose levels can themselves induce specific alterations in histone marks at the sites of genes encoding proteins important for cardiomyocyte survival and inflammation. For instance, high glucose has been proposed as being responsible for an HDAC1-dependent diminution in histone H4 acetylation at the insulin-like growth factor-1 receptor (IGF-1R) promoter that resulted in a decrease in IGF-1R expression accompanied by enhanced programmed cell death [117]. The same investigators followed this work up by demonstrating that transient exposure of H9c2 cells to high glucose caused decreased levels of the repressive H3K9me3 mark (and the H3K9 trimethylating enzyme SUV39H1) at the promoter region of the proinflammatory cytokine interleukin-6 (IL-6), resulting in persistent IL-6 upregulation [118].

**Table 5:** Articles implicating histone protein modifications in the development of heart failure

Disease or experimental context	Outcome	References
Human failing hearts	Pathological changes in gene expression associated with active histone marks: H3K27ac, H3K4me3, H3Kme1, H3K9ac, and H3K36me3	[115]
Human non-ischemic dilated cardiomyopathy	Reverse remodeling following left ventricular assist device (LVAD) implantation. Decreased H3K4me3, H3K9me2, and H3K9me3 in failing hearts, reversed with LVAD and associated with upregulation of SUV39H1 and downregulation of JMJD1A, JMJD2A, and JMJD2D	[172]
Cardiomyocyte-specific inducible G9a knockout mice and G9a inhibition in wild-type mice subjected to transverse aortic constriction (TAC)	G9a preserves cardiac function by demethylation of H3K9, through interaction with EZH2 and through forming a complex with the transcription factor, MEF2C	[173]
Uninephectomized db/db mice	Increased cardiac H3K23ac, H3K9ac, H3Ser10, phosphorylation, and H3K4me2, and decreased H3K9me2 favoring gene activation	[116]
Mice subjected to TAC surgery and human hypertrophic hearts	In response to pathological stress, cardiomyocytes express the nucleosome-remodeling factor, Brg1, G9a and the DNA methyltransferase, Dnmt3 which cause repression of α-MHC, marked by H3K9 and CpG methylation, impairing cardiac contraction	[174]
Post-myocardial infarction in rats	Treatment with c-kit^+^ cells exposed to the Class I HDAC inhibitor, mocetinostat increased acetylated H3 at the promoter regions of pluripotent and cardiac-specific genes and enhanced cardiac recovery	[175]
Mice subjected to TAC surgery	Epigenetic signature (325 of 1109 differentially expressed genes) characterized by mutual exclusion of activating (H3K9ac, H3K27ac, H3K4me3, and H3K79me2) and repressive (H3K9me2, H3K9me3, and H3K27me3) marks	[114]
TAC surgery in mice with cardiac-specific JMJD2A knockout, JMJD2A overexpressing mice, and human hypertrophic cardiomyopathy	JMJD2A promotes cardiac hypertrophy under pathological conditions; JMJD2A decreased H3K9me3 at the FHL1 promoter, facilitating cardiac hypertrophy	[105]
Treatment of Dahl salt-sensitive rats with the H3K9 methyltransferase inhibitor chaetocin	In heart failure, increased H3K9me3 at repetitive elements including intronic regions of mitochondria-related genes; chaetocin decreased H3K9me3, improved mitochondrial function and preserved cardiac contractility	[176]
Neonatal rat cardiomyocytes and ovariectomized wild-type and estrogen receptor β knockout mice administered angiotensin II	Angiotensin II stimulated H3 acetylation at the β-MHC promoter and this was prevented by 17β-estradiol or estradiol receptor β agonism, which repressed the prohypertrophic Class I HDAC, HDAC2 and de-repressed the antihypertrophic Class II HDACs, HDAC4 and HDAC5	[177]
Phenylephrine-induced cardiac hypertrophy in mice	The Chinese herbal extract, anacardic acid decreased binding of the HATs, p300, and PCAF and H3K9 acetylation at the MEF2A promoter and attenuated cardiac hypertrophy	[178]
Neonatal rat cardiomyocytes	Nuclear CaMKII activates cardiac gene transcription and promotes hypertrophy by phosphorylating H3Ser10	[109]
Human hearts with end-stage heart failure and TAC surgery in CaMKIIδ knockout mice	CaMKIIδ and H3Ser10 phosphorylation are increased in human failing hearts and CaMKIIδ knockout attenuates H3 serine 10 phosphorylation in TAC hearts	[108]
TAC surgery in rats and mice, exercise training in rats, cardiomyocyte-specific EHMT2 knockout, human cardiac tissue, and neonatal rat cardiomyocytes	Pathological hypertrophy is associated with loss of H3K9me2 and reexpression of fetal genes; pathological hypertrophy increases miR-217 which decreases expression of the H3K9 methylating enzymes, EHMT1 and EHMT2	[179]
TAC surgery in PTIP knockout mice	Global H3K4me3 levels are reduced in PTIP knockout mice which exhibited a maladaptive response to TAC surgery	[104]
Neonatal rat cardiomyocytes	Rosiglitazone increased H3Ser10 phosphorylation globally and at the ANP promoter and increased cardiomyocyte hypertrophy	[180]
Fructose-fed rats and H9c2 cells	Resveratrol deacetylated NF-κB-p65 and H3K9 and attenuated cardiac hypertrophy and oxidative stress through decreased NOX transcription	[119]
Cardiac mesenchymal stem cells from humans with Type 2 diabetes	Cardiac mesenchymal stem cells from humans with Type 2 diabetes have decreased proliferation and premature senescence associated with decreased H3Ser10 phosphorylation; decreased H3K9ac and H3K14ac; increased H3K9me3 and H3K27me3	[127]
H9c2 cells	High glucose caused an HDAC1-dependent diminution in H4ac at the IGF-1R promoter, decreasing IGF-1R expression and enhancing programmed cell death	[117]
H9c2 cells	Transient exposure to high glucose caused a persistent reduction of SUV39H1 and H3K9me3 at the IL-6 promoter	[118]
OVE26 mice	HDAC3 activity is increased in the hearts of Type 1 diabetic OVE26 mice; HDAC3 inhibition increased H3 acetylation at the DUSP5 promoter preventing DUSP5 downregulation and improving diabetes-induced cardiac dysfunction	[120]

Other studies have reported the effects of enzymes that can modify histone proteins in cardiac cells or experimental models. Because these enzymes may post-translationally modify histone and non-histone proteins, this table lists only studies in which specific histone changes have been reported

*LVAD* left ventricular assist device, *SUV39H1* suppressor of variegation 3–9 homolog 1, *JMJD* jumonji domain-containing, *TAC* transverse aortic constriction, *EZH2* enhancer of zeste homolog 2, *MEF* myocyte enhancer factor, *Dnmt3* DNA methyltransferase 3, *MHC* myosin heavy chain, *FHL1* four and a half LIM domains protein 1, *HDAC* histone deacetylase, *HAT* histone acetyltransferase, *PCAF* p300/CBP-associated factor, *CaMKII* calcium/calmodulin-dependent protein kinase II, *miR-217* microRNA 217, *EHMT* euchromatic histone-lysine *N*-methyltransferase, *PTIP* pax transcription activation domain-interacting protein, *ANP* atrial natriuretic peptide, *NF-κB* nuclear factor kappa-light-chain-enhancer of activated B cells, *NOX* NADPH oxidase, *IL-6* interleukin-6, *IGF-1R* insulin-like growth factor-1 receptor, *DUSP5* dual specificity phosphatase 5

In vivo, treatment of fructose-fed diabetic rats with resveratrol deacetylated both the p65 subunit of nuclear factor kappa-light-chain-enhancer of activated B cells (NF-κB) and H3K9, and attenuated cardiac hypertrophy and oxidative stress through downregulation of NADPH oxidase [119]. Separately, activity of the Class I HDAC, HDAC3 was found to be significantly increased in the hearts of Type 1 diabetic OVE26 mice, whereas selective HDAC3 inhibition improved diabetes-induced cardiac dysfunction [120]. In that study, HDAC3 inhibition led to the acetylation of histone H3 at the promoter region of the gene encoding the nuclear ERK1/2 phosphatase, dual specificity phosphatase 5 (DUSP5), preventing diabetes-associated DUSP5 downregulation, which the authors speculated was responsible for ERK1/2-driven cardiac dysfunction in diabetes [120]. A number of other studies have reported an improvement in the cardiac phenotype of diabetic rodents treated with pharmacological agents that influence the activity of HAT [121, 122] or HDAC enzymes [123–126]. However, because of the breadth of their enzymatic substrates, it is difficult to determine which, if any, of their effects are mediated through the post-translational modification of histone proteins.

With respect to histone modification changes that occur in people with diabetes, cardiac mesenchymal cells obtained from individuals with Type 2 diabetes have been characterized as having diminished H3Ser10 phosphorylation levels accompanied by decreased differentiation potential, reduced proliferation, and premature senescence [127]. This observation is significant, because it serves to highlight the dual functionality of H3Ser10 phosphorylation. On one hand, H3Ser10 phosphorylation associates with open chromatin and facilitates gene transcription during interphase. On the other hand, it marks highly condensed chromatin during mitosis, peaking during metaphase [128, 129], and it may, therefore, be used as a marker of cellular proliferation. Aside from these changes in histone phosphorylation, cardiac mesenchymal cells from individuals with Type 2 diabetes also exhibited a specific histone signature characterized by a predominance of histone marks associated with transcriptional repression (H3K9me3, H3K27me3, and H3K20me3) together with a decrease in histone marks typically associated with active chromatin (H3K9ac and H4K16ac) [127]. Moreover, treatment with a GNAT proactivator was able to restore H3K9ac and H4K16ac levels, and improve the proliferation and differentiation of cardiac mesenchymal cells derived from individuals with Type 2 diabetes [127].

## The role of histone protein modifications in pathogenetic processes important to the development of heart failure in diabetes but studied in non-cardiac cells

Although numerous studies have pointed to the importance of histone protein modifications in the development of heart failure, relatively few have examined their effects in heart failure in diabetes. This may be, at least in part, due to the limitations imposed by current models of cardiac dysfunction in diabetes. In particular, it is noteworthy that much of the current evidence indicating that histone protein post-translational modifications can affect cardiac function is derived from studies that have employed the TAC model of hypertrophic heart failure (Table 5). LVH is also a common predeterminant of heart failure in people with diabetes [130]. However, it is typically absent in rodent models of diabetes. For instance, the most widely studied model of diabetes and cardiac dysfunction is the streptozotocin-induced diabetes model, yet these animals do not develop cardiac hypertrophy [131] and the most commonly studied model of Type 2 diabetes is the db/db mouse [131], yet db/db mice appear to be relatively protected from cardiac dysfunction induced by TAC surgery [130]. Thus, the limitations of current experimental models may impede elucidation of the role of histone modifications in heart failure in diabetes. In this context, is it instead possible to derive clues as to their potential roles by examining the effects of histone post-translational modifications in other diabetes complications that are caused by similar pathogenetic processes?

### Fibrosis

Fibrosis is a common end process in heart failure, including in the diabetic setting [132]. Whereas little is known about the influence of histone protein modifications on cardiac fibrosis in diabetes, their effects on fibrosis of the diabetic kidney have been explored (reviewed in Ref. [133]). Here, we highlight two examples in which specific histone modifications have been found to promote fibrotic gene expression in renal cells. In a seminal report published in 2010, Sun and co-workers probed for changes in histone methylation marks at the promoter regions of genes encoding extracellular matrix proteins in rat glomerular mesangial cells exposed to the profibrotic growth factor, transforming growth factor-β1 (TGF-β1) [126]. The investigators found increased levels of chromatin marks associated with active genes (i.e., H3K4me1-3, accompanied by increased expression and recruitment of the H3K4 methyltransferase SET7/9) and decreased levels of marks associated with gene repression (i.e., H3K9me2 and H3K9me3), whereas treatment of high glucose exposed cells with a neutralizing TGF-β1 antibody prevented these histone changes [126]. Separately, myocardin-related transcription factor-A (MRTF-A) is a serum response factor cofactor that promotes cardiac myofibroblast activation and fibrosis [134]. In diabetic mice, deletion of MRTF-A attenuated renal tubulointerstitial fibrosis and was accompanied by the loss of histone modifications indicative of transcriptional activation (i.e., H3K18ac, H3K27ac, and H3K4me3) [135]. These effects, in renal epithelial cells exposed to high glucose, were attributed to the recruitment by MRTF-A of both the histone acetyltransferase, p300, and a component of the H3K4 methyltransferase complex, WD repeat-containing protein 5 to the promoter regions of target fibrotic genes [135].

### Advanced glycation end products

Advanced glycation end products are formed when nonenzymatic reactions take place between reducing sugars and free amino groups of proteins, lipids, or nucleic acids [136]. Because they are stable and long lasting, AGEs have been implicated in the development and progression of many of the long-term complications of diabetes [137], including heart failure [132]. AGEs may directly modify histone proteins, and this may directly affect chromatin structure and gene expression [138, 139]. The effects of AGEs may also themselves be influenced by histone protein modifications. For instance, in monocytes, the induction of inflammatory genes by ligands of the receptor for advanced glycation end products (RAGE) was attenuated by knockdown of SET7/9 [140].

### Oxidative stress

Studies have demonstrated a bidirectional association between histone modifications and oxidative stress in diabetes, whereby, on one hand, reactive oxygen species (ROS) may influence the formation of new histone modifications under high glucose conditions, and, on the other hand, histone modifications may influence the development of oxidative stress. As an illustration of how ROS may influence histone modifications, El-Osta and co-workers demonstrated the effects of transient exposure of aortic endothelial cells to high glucose on histone changes at the promoter region of the gene encoding the p65 subunit of the proinflammatory nuclear factor, NF-κB [141]. They found that high glucose caused persistent p65 upregulation that was mediated by SET7-induced histone H3K4 monomethylation [141]. This persistent upregulation of p65 was prevented by overexpression of either uncoupling protein-1 or manganese superoxide dismutase (MnSOD), which both prevent high glucose-induced superoxide accumulation, or by overexpression of glyoxalase 1, which metabolizes methylglyoxal a precursor of AGEs [141]. In contrast, in rat retinal endothelial cells, regulation of the gene encoding MnSOD is itself associated with specific chromatin modifications under diabetic conditions (i.e., H4K20me3 and H3K9ac) [142]. Our own work on histone modifications and oxidative stress in diabetes has focused on the regulation of enzymatic antioxidant repair mechanisms. Thioredoxin-interacting protein (TxnIP) is a glucose-regulated inhibitor of the endogenous antioxidant enzyme, thioredoxin. TxnIP is upregulated in both the diabetic kidney [143] and the diabetic heart [144], and it predisposes to diabetes-associated oxidative damage [143, 144]. In mouse glomerular podocytes, depletion of the histone H3K27 trimethylating enzyme enhancer of zeste homolog 2 (EZH2) was accompanied by de-repression of TxnIP and augmented oxidative injury [145]. Interestingly, however, in these studies, we did not find the repressive H3K27me3 mark at the mouse TxnIP promoter; rather, EZH2 depletion caused loss of H3K27me3 at the promoter region of the gene encoding the transcription factor Pax6 associated with de-repression of Pax6 which subsequently bound to the TxnIP promoter controlling expression of its gene product [145].

### Endothelial dysfunction

TxnIP may itself also influence endothelial dysfunction in diabetes by inducing histone modifications. In rat retinal endothelial cells, for example, RAGE activation upregulated cyclooxygenase-2 (COX-2) in a TxnIP-dependent manner and TxnIP overexpression caused an increase in the H3K9ac mark (associated with transcriptional activation) and a decrease in the H3K9me3 mark (associated with transcriptional repression) at the proximal COX-2 promoter [146]. p66^Shc^ is an adaptor protein that contributes to mitochondrial ROS generation by accepting electrons from cytochrome c. In endothelial cells, the class III HDAC, SIRT1 binds to the p66^Shc^ promoter region, causing a decrease in histone H3 acetylation, repressing p66^Shc^ and protecting against hyperglycemia-induced endothelial dysfunction [147]. In individuals with Type 2 diabetes, intensive glycemic control did not improve brachial artery flow-mediated dilatation and, associated with this persistent abnormality in endothelial function, there was persistent downregulation of SIRT1 and of histone H3 acetylation at the p66^Shc^ promoter (along with DNA hypomethylation) [148].

### Inflammation

A number of examples already cited above have explored the contributions of histone protein modifications to the long-term complications of diabetes in the context of inflammation. Elsewhere, in glomerular podocytes, the class III HDAC, SIRT6 limits inflammation and deacetylates H3K9 at the promoter regions of the Notch ligands, Notch1 and Notch4 [149]; the proinflammatory phenotype of macrophages in Type 2 diabetes has been attributed to a Jmjd3-mediated loss of the repressive H3K27me3 mark at the promoter region of the gene encoding IL-12 [150]; and vascular smooth muscle cells (VSMCs) from db/db mice exhibited loss of the repressive H3K9me3 histone mark at the promoter regions of key proinflammatory genes, as did human VSMCs exposed to high glucose [151].

### Glucose metabolism, fatty acid utilization, and small vessel disease

The roles that epigenetic processes play in glucose metabolism and fatty acid utilization have recently been reviewed elsewhere [152–154]. With respect to small vessel disease, histone protein changes have been observed at the gene encoding matrix metalloproteinase-9 (MMP-9), which augments retinal capillary cell programmed cell death. More particularly, Zhong and co-workers found decreased levels of the repressive H3K9me2 mark and increased levels of the activating H3K9ac mark at the MMP-9 promoter in high glucose exposed retinal endothelial cells [155]. This effect was attributed by the authors to diabetes-induced increases in the expression and activity of LSD1 which demethylates H3K9me2, freeing up H3K9 for acetylation and, in turn, facilitating the recruitment of p65, upregulating MMP-9, and predisposing to mitochondrial damage and cell death [155].

**Fig. 3:**
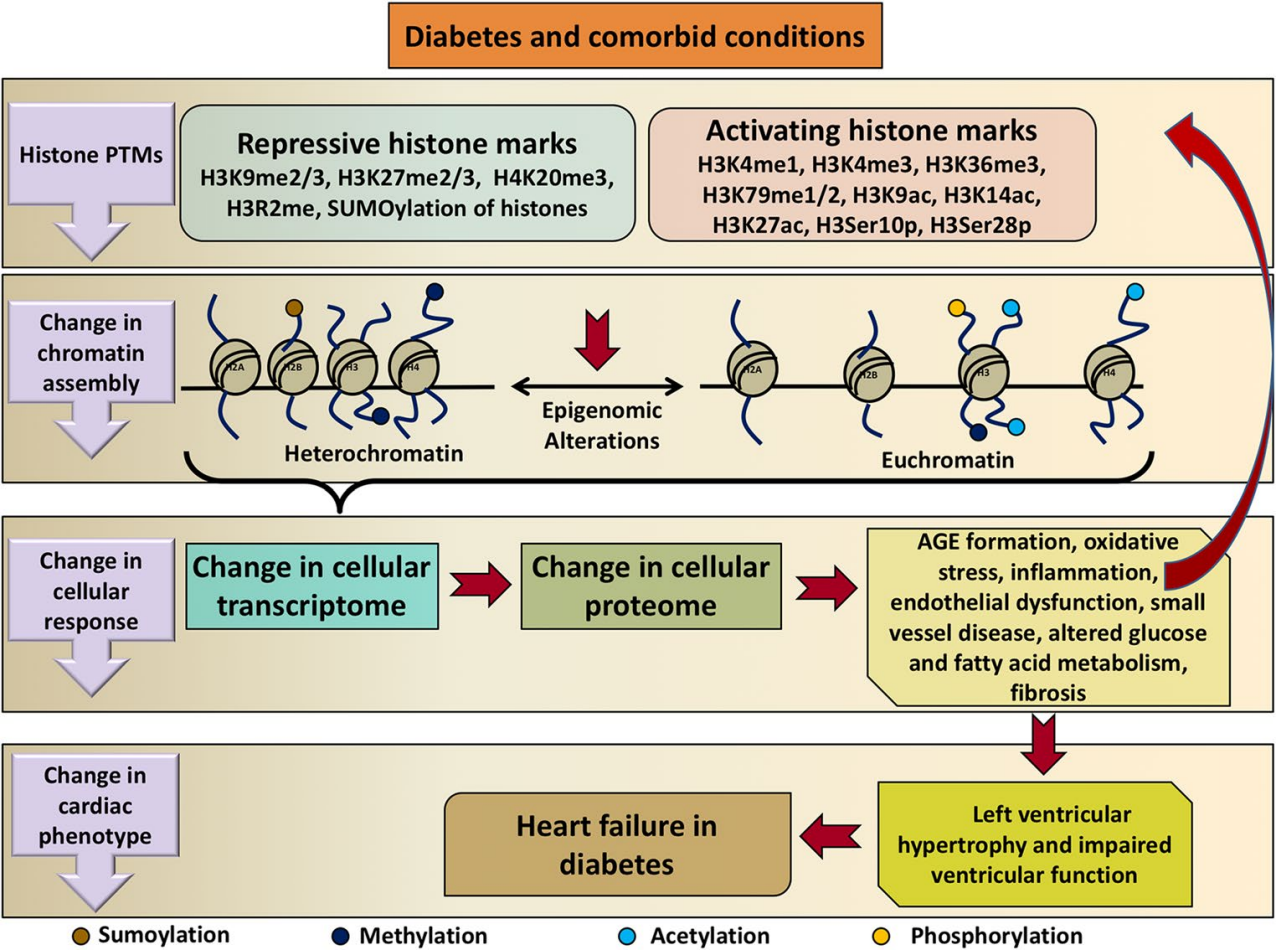
Schematic illustration of the roles that histone protein post-translational modifications may play in the development of heart failure in diabetes. Diabetes and its comorbidities cause changes in repressive and activating histone marks that alter chromatin assembly. This in turn affects the cellular transcriptome and proteome promoting cellular injury processes in diabetes. These processes can themselves affect histone protein modifications. The result is a change in cardiac phenotype leading to left ventricular hypertrophy and dysfunction and thus heart failure in diabetes. *PTM* post-translational modification, *H3R2me* methylation of arginine 2 on histone H3, *H3Ser10p* phosphorylation of serine residue 10 on histone H3, *H3Ser28p* phosphorylation of serine residue 28 on histone H3, *AGE* advanced glycation end product

In summary, a body of literature evidence exists attesting to the roles that histone protein post-translational modifications play in the development of heart failure and a comparable body of literature exists attesting to their contributions to end-organ injury in diabetes. Evidence is likewise beginning to emerge linking histone modifications specifically to the development of heart failure in diabetes. The putative mechanisms through which this may occur are summarized in Fig. 3. It is noteworthy, however, that one of the defining characteristics of histone modifications is that they are lineage-specific [156]. Thus, caution should be taken in extrapolating findings from other model systems and the determination of the extent to which histone modifications do contribute to the development of heart failure in diabetes requires further direct experimentation.

## Future directions: therapeutically targeting histone-modifying enzymes in the clinic

Whereas the understanding of the fundamental mechanisms of disease is creditable in its own right, the evolving diabetes pandemic and the grim prognosis of those individuals who develop heart failure in diabetes demand our earnest efforts to seek out new treatment opportunities. Thus, what evidence exists that histone protein modifications can be amenable to therapeutic manipulation in patients? A number of therapies that target histone-modifying enzymes have received regulatory authority approval or are undergoing clinical trial evaluation (Table 6). In most cases, these agents are currently undergoing evaluation, or have been approved, for the treatment of various malignancies, particularly hematological malignancies.

**Table 6:** Approved therapies and therapies under clinical trial evaluation whose mechanism of action involves the inhibition of histone-modifying enzymes

Therapy	Class	Latest stage of development	Indication or National Clinical Trial identifier number
Vorinostat	HDAC inhibitor	FDA approved	Cutaneous T cell lymphoma
Romidepsin	HDAC inhibitor	FDA approved	Cutaneous T-cell lymphoma and other peripheral T-cell lymphomas
Belinostat	HDAC inhibitor	FDA approved	Peripheral T-cell lymphoma
Panobinostat	HDAC inhibitor	FDA approved	Multiple myeloma
Chidamide	HDAC inhibitor	Approved in China	Relapsed or refractory T-cell lymphoma
Entinostat	HDAC inhibitor	Phase 3	NCT02115282
Tazemetostat	EZH2 inhibitor	Phase 2	NCT018975751, NCT02875548, NCT02601950
Givinostat	HDAC inhibitor	Phase 2	NCT01761968
Mocetinostat	HDAC inhibitor	Phase 2	NCT0205660, NCT02954991
Tinostamustine	First-in-class alkylating HDAC inhibitor	Phase 1/2	NCT03345485
INCB059872	LSD1 inhibitor	Phase 1/2	NCT02712905
DS-3201b	EZH1/EZH2 dual inhibitor	Phase 1	NCT02732275
CXD101	HDAC inhibitor	Phase 1	NCT01977638
MPT0E028	HDAC inhibitor	Phase 1	NCT02350868
CPI-1205	EZH2 inhibitor	Phase 1	NCT02395601

If the therapeutic manipulation of histone protein modifications is going to find a place in the treatment of heart failure in diabetes advances need to be made at both the fundamental and pharmacological levels. As has already been highlighted, evidence exists that histone protein post-translational modifications are awry in heart failure and in other long-term complications of diabetes. However, much needs to be done to better understand their relative contributions to the development of heart failure in diabetes. This will require animal models that better recapitulate the human disease state and a recognition that humans and rodents will likely differ in their disease-specific epigenomes [157]. At the pharmacological level, the therapies that are most advanced in their clinical development are inhibitors of HDAC enzymes and inhibitors of the H3K27 trimethylating enzyme, EZH2 (Table 6). As already emphasized, at least in the former case, given the breadth of their potential substrates, the biologic effects of HDAC inhibitors in the clinic cannot be assumed to be mediated solely by the acetylation of histone proteins. Furthermore, these agents may have adverse effect profiles that, whilst tolerable in the cancer setting, are unacceptable for the treatment of chronic disease. For instance, the most common adverse effects of the HDAC inhibitor vorinostat (occurring with an incidence ≥ 20%) are diarrhea, nausea, anorexia, fatigue, thrombocytopenia, and dysgeusia [158]. Finally, most of the currently utilized HDAC inhibitors lack HDAC specificity. Vorinostat, for instance, inhibits Class I HDACs (HDAC1, HDAC2, and HDAC3) and Class II HDACs (HDAC6) at low nanomolar concentrations (< 86 nM) [159]. Whereas other HDAC inhibitors may inhibit solely Class I HDACs (e.g., mocetinostat and entinostat) [160, 161], the development of isoform-specific agents is still in its relative infancy [162–165]. Whether it will be possible to develop agents with sufficient tolerability and with sufficient specificity that they can be used for the treatment of complex chronic diseases, such as heart failure, remains to be seen.

## Summary

It is 15 years, since Dr. David Bell described heart failure as “the frequent, forgotten, and often fatal complication of diabetes” [4]. Although the results of recent outcome trials assure that heart failure may, perhaps, no longer be the forgotten complication of diabetes, it remains frequent and often fatal. Histone protein modifications have emerged as pivotal players in both the development of diabetes-associated injury to other organ systems and in heart failure that is not caused by diabetes, especially hypertrophic heart failure which is a common occurrence in patients with diabetes. Histone protein post-translational modifications are amenable to therapeutic manipulation and there is a rapidly proliferating armamentarium of small molecule pharmaceuticals that alter these processes and that are under investigation in other disease settings. It remains to be seen whether altered histone protein modifications may be amenable enough or whether any of the existing or future tools will be specific enough or tolerable enough to improve outcomes for the millions of individuals currently affected by heart failure in diabetes.

## Abbreviations

HR: Hazard ratio
CI: Confidence interval
USD: United States dollars
CHARM: Candesartan in Heart failure: Assessment of Reduction in Mortality and Morbidity
EF: Ejection fraction
HFrEF: Heart failure with reduced ejection fraction
HFpEF: Heart failure with preserved ejection fraction
AGE: Advanced glycation end product
ECG: Electrocardiogram
LVH: Left ventricular hypertrophy
ACE: Angiotensin converting enzyme
ARB: Angiotensin receptor blocker
ARNI: Angiotensin receptor–neprilysin inhibitor
GLP-1: Glucagon-like peptide-1
DPP-4: Dipeptidyl peptidase-4
SAVOR: Saxagliptin Assessment of Vascular Outcomes Recorded in Patients with Diabetes Mellitus
TIMI: Thrombolysis in Myocardial Infarction
EXAMINE: Examination of Cardiovascular Outcomes with Alogliptin versus Standard of Care
SGLT2: Sodium–glucose cotransporter 2
CANVAS: Canagliflozin Cardiovascular Assessment Study
HAT: Histone acetyltransferase
HDAC: Histone deacetylase
KAT: Lysine acetyltransferase
KMT: Lysine methyltransferase
KDM: Lysine demethylase
KDAC: Lysine deacetylase
MLL: Mixed lineage leukemia or myeloid/lymphoid leukemia
JMJD: Jumonji domain-containing
5mC: 5-Methylcytosine
5hmC: 5-Hydroxymethylcytosine
MECP2: Methyl CpG binding protein 2
TET: Ten–eleven translocation
Dnmt: DNA methyltransferase
IRF1: Interferon regulatory factor 1
FOXO3: Forkhead box O3 (FOXO3),
pRb: Retinoblastoma protein (pRB)
STAT3: Signal transducer and activator of transcription 3
PCAF: p300/CBP-associated factor
GNAT: Gcn5-related *N*-acetyltransferase
CBP: CREB-binding protein
MEF2: Myocyte enhancer factor 2
SAM: *s*-Adenosylmethionine
LSD: Lysine-specific demethylase
FAD: Flavin adenosine dinucleotide
CoREST: Restin corepressor
PTIP: Pax transcription activation domain-interacting protein
TAC: Transverse aortic constriction
CaMKII: Calcium/calmodulin-dependent protein kinase II
SUMO: Small ubiquitin-l modifier
NF-κB: Nuclear factor kappa-light-chain-enhancer of activated B cells
DUSP5: Dual specificity phosphatase 5
TGF-β1: Transforming growth factor-β1
MRTF-A: Myocardin-related transcription factor-A
RAGE: Receptor for advanced glycation end products
ROS: Reactive oxygen species
MnSOD: Manganese superoxide dismutase
TxnIP: Thioredoxin-interacting protein
EZH: Enhancer of zeste homolog
COX-2: Cyclooxygenase-2
IL: Interleukin
MMP-9: Matrix metalloproteinase-9
ELP3: Elongator protein complex 3
MOZ: Monocytic leukemia zinc finger protein
MORF: Monocyte leukemia zinc finger protein-related factor
HBO1: Histone acetyltransferase binding to ORC-1
CLOCK: Clock circadian regulator
SRC1: Steroid receptor coactivator-1
TIF2: Transcriptional mediators/intermediary factor 2
SIRT: Silent mating-type information regulation 2 homolog
SMYD: SET and MYND domain-containing
SETMAR: SET domain and mariner transposase fusion protein
PRDM: PR domain
SUV39H: Suppressor of variegation 3–9 homolog
EHMT: Euchromatic histone-lysine *N*-methyltransferase
GLP: G9a-like protein
ESET: ERG-associated protein with SET domain
SETDB: SET domain bifurcated
SETD: SET domain-containing
NSD: Nuclear receptor-binding SET domain protein
DOT1L: DOT1-like histone-lysine methyltransferase
JHDM: Jumonji C domain-containing histone demethylase
FBXL: F-box and leucine-rich repeat protein
JARID: Jumonji/ARID domain-containing protein
RBP2: Retinoblastoma-binding protein 2
SMCX: Smcx homolog, X chromosome
SMCY: SMC homolog, Y chromosome
GASC1: Gene amplified in squamous cell carcinoma 1
PHF: PHD finger protein
UTX: Ubiquitously transcribed X chromosome tetratricopeptide repeat protein
LVAD: Left ventricular assist device
MHC: Myosin heavy chain
FHL1: Four and a half LIM domains protein 1
miR-217: MicroRNA 217
ANP: Atrial natriuretic peptide
NOX: NADPH oxidase
IGF1R: Insulin-like growth factor-1 receptor
